# Comparison between Karyotyping-FISH-Reverse Transcription PCR and RNA- Sequencing-Fusion Gene Identification Programs in the Detection of *KAT6A*-*CREBBP* in Acute Myeloid Leukemia

**DOI:** 10.1371/journal.pone.0096570

**Published:** 2014-05-05

**Authors:** Ioannis Panagopoulos, Synne Torkildsen, Ludmila Gorunova, Anne Tierens, Geir E. Tjønnfjord, Sverre Heim

**Affiliations:** 1 Section for Cancer Cytogenetics, Institute for Cancer Genetics and Informatics, The Norwegian Radium Hospital, Oslo University Hospital, Oslo, Norway; 2 Centre for Cancer Biomedicine, Faculty of Medicine, University of Oslo, Oslo, Norway; 3 Department of Hematology, Oslo University Hospital, Oslo, Norway; 4 Department of Pathology, The Norwegian Radium Hospital, Oslo University Hospital, Oslo, Norway; 5 Faculty of Medicine, University of Oslo, Oslo, Norway; European Institute of Oncology, Italy

## Abstract

An acute myeloid leukemia was suspected of having a t(8;16)(p11;p13) resulting in a *KAT6A*-*CREBBP* fusion because the bone marrow was packed with monoblasts showing marked erythrophagocytosis. The diagnostic karyotype was 46,XY,add(1)(p13),t(8;21)(p11;q22),der(16)t(1;16)(p13;p13)[9]/46,XY[1]; thus, no direct confirmation of the suspicion could be given although both 8p11 and 16p13 seemed to be rearranged. The leukemic cells were examined in two ways to find out whether a cryptic *KAT6A*-*CREBBP* was present. The first was the “conventional” approach: G-banding was followed by fluorescence in situ hybridization (FISH) and reverse transcription PCR (RT-PCR). The second was RNA-Seq followed by data analysis using FusionMap and FusionFinder programs with special emphasis on candidates located in the 1p13, 8p11, 16p13, and 21q22 breakpoints. FISH analysis indicated the presence of a *KAT6A/CREBBP* chimera. RT-PCR followed by Sanger sequencing of the amplified product showed that a chimeric *KAT6A-CREBBP* transcript was present in the patients bone marrow. Surprisingly, however, *KATA6A-CREBBP* was not among the 874 and 35 fusion transcripts identified by the FusionMap and FusionFinder programs, respectively, although 11 sequences of the raw RNA-sequencing data were *KATA6A-CREBBP* fragments. This illustrates that although many fusion transcripts can be found by RNA-Seq combined with FusionMap and FusionFinder, the pathogenetically essential fusion is not always picked up by the bioinformatic algorithms behind these programs. The present study not only illustrates potential pitfalls of current data analysis programs of whole transcriptome sequences which make them less useful as stand-alone techniques, but also that leukemia diagnosis still relies on integration of clinical, hematologic, and genetic disease features of which the former two by no means have become superfluous.

## Introduction

The chromosome aberration t(8;16)(p11;p13) was first described in 1983 in an infant in whom the leukemic cells displayed prominent hemophagocytosis [1]. The recurrence of t(8;16)(p11;p13) in acute myeloid leukemia (AML) was independently established in 1987 by three groups. Bernstein et al [2] reported two infants with AML carrying the t(8;16)(p11;p13). Heim at al [3] described three cases of AML, two teenagers and one infant, with the t(8;16) as the sole chromosome abnormality. Lai et al [4] reported three more cases of t(8;16)-positive AML with additional structural chromosome aberrations present in two of them. Monocytic differentiation and phagocytosis were distinctive features of all the patients [2], [3], [4]. In the Mitelman Database of Chromosome Aberration and Gene Fusions in Cancer, there are now 116 cases of AML carrying the t(8;16)(p11;p13) chromosome abnormality (http://cgap.nci.nih.gov/Chromosomes/Mitelman, database last updated on August 14, 2013).

AML with t(8;16)(p11;p13) is now recognized as a distinct disease entity characterized by monocytic differentiation of the leukemic cells and marked erythrophagocytosis [5], [6], frequent skin involvement, and a tendency to develop diffuse intravascular coagulation. It often occurs at a young age and the response to treatment is poor resulting in short survival [5], [7].

The translocation t(8;16)(p11;p13) disrupts *KAT6A* (also known as *MOZ* and *MYST3*) on 8p11 and *CREBBP* (also named *CBP*) on 16p13 resulting in a fusion of the two genes [8], [9]. Although genomic rearrangements of *KAT6A* and *CREBBP* were repeatedly detected using fluorescence in situ hybridization (FISH) and Southern blot methodologies [8], [9], [10], attempts to amplify and further analyze chimeric *KAT6A*-*CREBBP* and *CREBBP-KAT6A* transcripts using reverse transcriptase-PCR (RT-PCR) analysis were long unsuccessful [8], [9], [10]. Various explanations such as low expression and/or instability of the chimeric transcripts were proposed [9]. Nevertheless, an RT-PCR strategy was developed to detect the *KAT6A*-*CREBBP* as well as *CREBBP-KAT6A* fusions [11] and two types of *KAT6A*-*CREBBP* fusion transcripts were identified: Type 1 was an in-frame transcript between codon 1117 of *KAT6A* and codon 29 of *CREBBP*, while type 2 was an out-of-frame transcript between exon codon 1117 of *KAT6A* and codon 267 of *CREBBP*. Subsequent studies confirmed the presence of *KAT6A*-*CREBBP* and *CREBBP-KAT6A* fusions, and it was shown that type 1 was the most frequent *KAT6A*-*CREBBP* fusion transcript [12], [13], [14]. Moreover, new *KAT6A*-*CREBBP* fusion transcript variants were described [15], [16] and real time PCR methodology (RT-PCR) was developed to monitor minimal residual disease status throughout the entire course of the treatment [17].

Recently, RNA-sequencing (RNA-seq, also known as whole transcriptome sequencing) was shown to be an efficient tool in the detection of fusion genes in cancer [18] and has created euphoria among those working with cancer fusion genes. The methodology is in principle simple: extracted RNA from cancer cells is massively sequenced, and then the raw data are analyzed with one or more programs specifically dedicated to the task of detecting fusion transcripts such as FusionMap and FusionFinder [19], [20]. However, it suffers from the shortcoming of identifying as “fusion genes” also many technical and perhaps also clinical “false positives,” thus making the assessment of which fusions are important and which are noise extremely difficult. We and others have used combinations of cytogenetics and RNA-seq to detect the “primary” fusion genes of neoplasms carrying only one or a few chromosomal rearrangements. This approach was used to identify the *WWTR1-CAMTA1* and *YWHAE-FAM22A/B* chimeric genes in epithelioid hemangioendothelioma and high-grade endometrial stromal sarcomas, respectively [21], [22], *ZC3H7-BCOR* in endometrial stromal sarcomas [23], *IRF2BP2-CDX1* in a mesenchymal chondrosarcoma [24], and *EWSR1-YY1* in a subset of mesotheliomas [25]. In hematologic malignancies, an *NFIA-CBFA2T3* (*NFIA* is located in 1p31) chimeric transcript was found in an acute erythroid leukemia with the translocation t(1;16)(p31;q24) [26], the *ZMYND8-RELA* fusion was detected in a congenital acute erythroid leukemia carrying a t(11;20)(p11;q13) translocation [27], and a cryptic *FUS/ERG* fusion gene was found in an acute myeloid leukemia with a rather complex karyotype [28].

We here describe a case of AML in which two translocations corresponding to a der(16)t(1;16)(p13;p13) and a t(8;21)(p11;q22) were found in the bone marrow cells. The patient had morphological features that invoked the suspicion of a t(8;16)(p11;p13) with *KAT6A*-*CREBBP* fusion in spite of the fact that the breakpoints suggested various other candidate genes. We therefore studied the patient's leukemic cells in two different ways looking for *KAT6A*-*CREBBP*: “conventionally” by karyotyping followed by FISH followed by RT-PCR, and in the modern manner using RNA-seq and programs specific for fusion genes.

## Materials and Methods

### Ethics Statement

The study was approved by the Regional Committee for Medical Research Ethics (Regional komité for medisinsk forskningsetikk Sør-Øst, Norge, http://helseforskning.etikkom.no). Written informed consent was obtained from the patient prior to his death. The ethics committee approval included a review of the consent procedure and all patient information has been anonymized and de-identified.

### Case history

**Figure 1:**
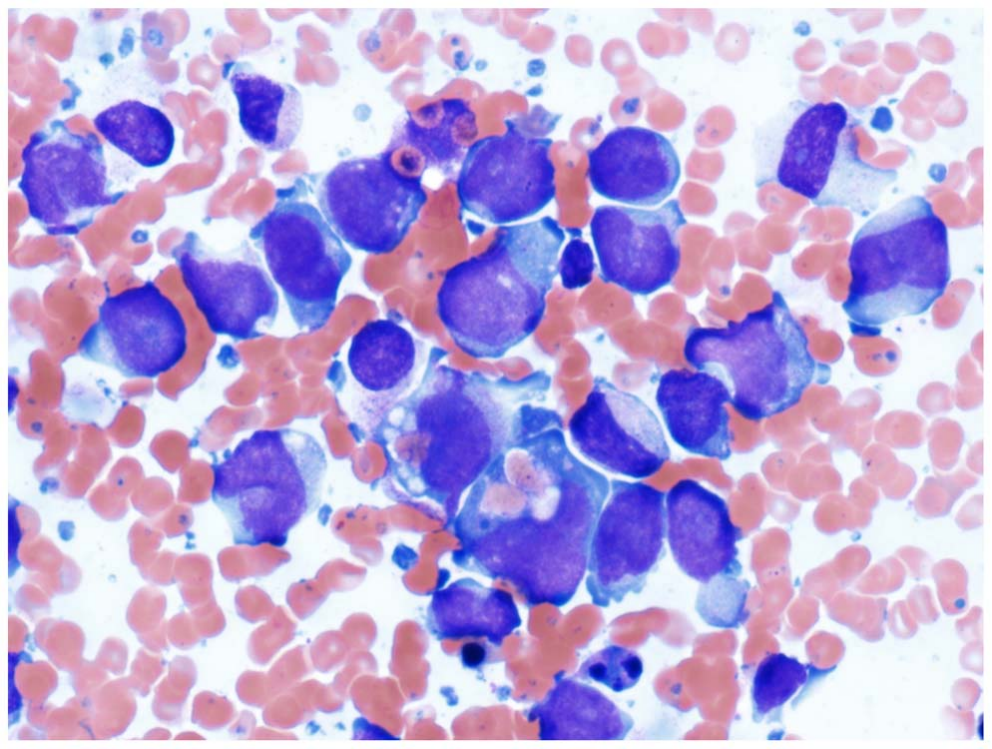
Bone marrow smear showing intermediate to large blasts with finely dispersed chromatin with variably abundant cytoplasm, vacuolization and phagocytosis of red blood cells (Wright-Giemsa 400 x).

A 30 years old male was transferred to our institution with a preliminary diagnosis of acute myeloid leukemia. He presented with fever and lower back pain radiating to the left lower limb. The clinical examination was unremarkable except for the presence of gingival petecchiae. Gingival hyperplasia was not noted. Blood analysis revealed a severe thrombocytopenia and an elevated CRP and LDH, but all other parameters were normal. Magnetic resonance investigation (MRI) of the columna demonstrated a paramedian prolapse between the L5/S1 vertebrae. A chest radiography showed bilateral infiltrations in the lower pulmonary lobes. Examination of a bone marrow aspirate showed that normal hematopoiesis was replaced by intermediate to large monoblasts, often with prominent vacuolization and ingested red blood cells (Figure 1). Immunophenotypic analysis confirmed the monocytic origin of the blasts that were positive for HLA-DR antigens, CD15, CD13, CD33, and cyMPO. Molecular genetic analysis was negative for *RUNX1-RUNX1T1* and *CBFB-MYH11* fusion transcripts as well as *FLT3* and *NPM1* mutations. The patient received induction therapy for AML with daunorubicin 90 mg/m2 day 1–3 and cytarabin 200 mg/m2 day 1–7 after which he went into morphologic remission. He then received two cycles of consolidation with high dose cytarabin (3 g/m2×2 per day, day 1, 3 and 5). One month later, however, he relapsed. He now received reinduction treatment with M5A5E5 (Amsakrin 150 mg/m2 day 1–3, Cytarabin 200 mg/m2/24 hours and Etoposide 110 mg/m2 daily for 5 days). Although morphologic remission was obtained, complete hematological recovery was not achieved 2 months after re-induction treatment.The consolidation treatment M3A5E3 was therefore given with an intent to proceed to allogenic stem cell transplantation, but the patient died 3 weeks later because of liver failure and an acute abdomen caused by ischemic infarcts of the intestine and liver.

### G-banding and FISH

Bone marrow cells were cytogenetically investigated by standard methods. Chromosome preparations were made from metaphase cells of a 24-hours culture, G-banded using Leishman stain, and karyotyped according to ISCN 2009 guidelines [29]. FISH analysis was performed on metaphase plates.

BAC clones were retrieved from the Human genome high-resolution BAC re-arrayed clone set (the “32k set”; BACPAC Resources, http://bacpac.chori.org/pHumanMinSet.htm). The “32k set” is mapped on the UCSC Genome browser on Human May 2004 (NCBI/hg17) assembly. Mapping data for the 32k human rearray are available in an interactive web format (http://bacpac.chori.org/pHumanMinSet.htm, from the genomic rearrays page) and are obtained by activation of the ucsc browser track for the hg17 UCSC assembly from the “32k set” homepage (http://bacpac.chori.org/genomicRearrays.php). The BAC clones were selected according to physical and genetic mapping data on chromosomes 8 and 16 (see below) as reported on the Human Genome Browser at the University of California, Santa Cruz website (May 2004, http://genome.ucsc.edu/). In addition, FISH mapping of the clones on normal controls was performed to confirm their chromosomal location. The clones used were RP11-619A23 (chr16:3660077-3854572) and RP11-95J11 (chr16:3800375-3965511) mapping to 16p13.3 and which both contain the entire *CREBBP* gene (red), and RP11-642I24 (chr8:41795493-41975651) and RP11-589C21 (chr8:41992859-42155379) mapping to 8p11.21 for *KAT6A* (green). DNA was extracted and probes were labelled and hybridized according to Abbott Molecular recommendations (http://www.abbottmolecular.com/home.html). Chromosome preparations were counterstained with 0.2 µg/ml DAPI and overlaid with a 24×50 mm^2^ coverslip. Fluorescent signals were captured and analyzed using the CytoVision system (Applied Imaging, Newcastle, UK).

### RT-PCR analyses

Total RNA was extracted from the patients bone marrow at the time of diagnosis using Trizol reagent according to the manufacturer's instructions (Invitrogen, Life Technologies, Oslo, Norway) and used for both RT-PCR and RNA-Seq analyses. For RT-PCR, one µg of total RNA was reverse-transcribed in a 20 µL reaction volume using iScript Advanced cDNA Synthesis Kit for RT-qPCR according to the manufacturer's instructions (Bio-Rad Laboratories, Oslo, Norway). The cDNA was diluted to 30 ng equivalent of RNA/µL and 2 µL were used as templates in subsequent PCR assays. The 25 µL PCR volume contained 12.5 µL Premix Ex Taq DNA Polymerase Hot Start Version (Takara Bio Europe/SAS, Saint-Germain-en-Laye, France), 2 µL of diluted cDNA, and 0.2 µM of each of the primers, the forward MOZ-3558F (5′-GAG GCC AAT GCC AAG ATT AGA AC-3′) and the reverse primer CBP-431R (5′-GTT GAT ACT AGA GCC GCT GCC TC-3′). The PCR was run on a C-1000 Thermal cycler (Bio-Rad Laboratories) with an initial denaturation at 94°C for 30 sec, followed by 35 cycles of 7 sec at 98°C, 30 sec at 55°C and 1 min at 72°C, and a final extension for 5 min at 72°C. Four µL of the PCR products were stained with GelRed (Biotium, VWR International, Oslo, Norway), analyzed by electrophoresis through 1.0% agarose gel, and photographed. The remaining PCR products were purified using the NucleoSpin Gel and PCR Clean-up kit (Macherey-Nagel, VWR International, Oslo, Norway) and sequenced at GATC Biotech (Germany, http://www.gatc-biotech.com/en/home.html). The BLAST software (http://blast.ncbi.nlm.nih.gov/Blast.cgi) was used for computer analysis of sequence data.

### RNA-sequencing (RNA-Seq)

Three µg of the total RNA extracted from the patients bone marrow at the time of diagnosis and used for RT-PCR analysis were sent for high-throughput paired-end RNA-sequencing at the Genomics Core Facility, The Norwegian Radium Hospital (http://genomics.no/oslo/). The Illumina software pipeline was used to process image data into raw sequencing data and only sequence reads marked as “passed filtering” were used in the downstream data analysis. A total of 53 million reads were obtained. The FASTQC software was used for quality control of the raw sequence data (http://www.bioinformatics.babraham.ac.uk/projects/fastqc/). Two softwares were used for the discovery of fusion transcripts: FusionMap [30] (release date 2012-04-16) together with the pre-built Human B37 and RefGene from the FusionMap website (http://www.omicsoft.com/fusionmap/) and FusionFinder [31]. In addition, the “grep” command (http://en.wikipedia.org/wiki/Grep) was used to search the fastq files of the sequence data (http://en.wikipedia.org/wiki/FASTQ_format).

## Results

### G-banding and FISH

#### G-banding analysis yielded the diagnostic karyotype

**Figure 2:**
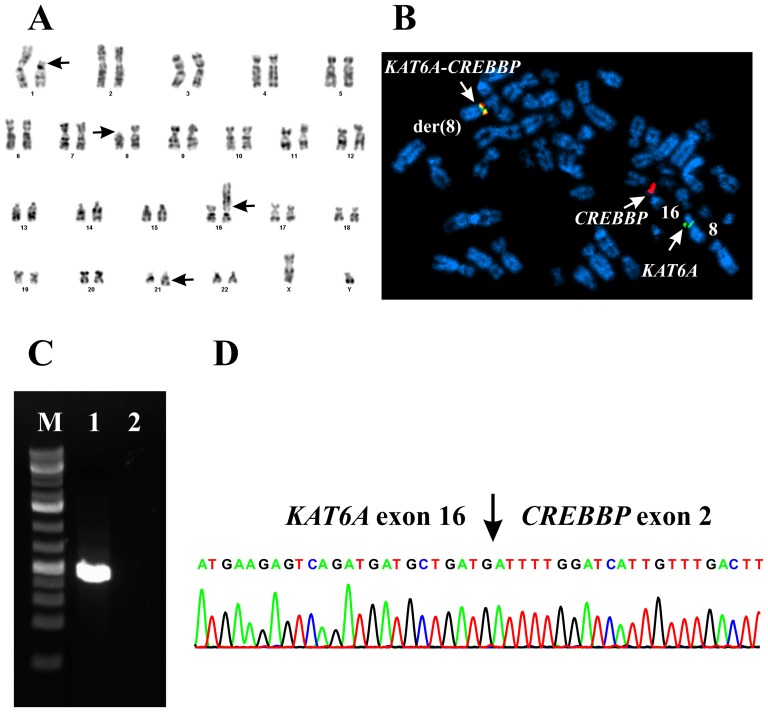
Cytogenetic, FISH and RT-PCR analyses. A) Karyotype at diagnosis showing the chromosome aberrations add(1)(p13), t(8;21)(p11;q22), and der(16)t(1;16)(p13;p13); breakpoint positions are indicated by arrows. B) Co-hybridization FISH analysis with the probes RP11-619A23/RP11-95J11 (red, for *CREBBP*) and RP11-642I24/RP11-589C21 (green, for *KAT6A*). A fusion signal of the *KAT6A* and *CREBBP* BACs is detected on the derivative chromosome 8, indicating the presence of a *KAT6A/CREBBP* chimera. C) Amplification of a 352 bp cDNA fragment using the primers MOZ-3558F and CBP-431R (lane 1); M, 1 Kb DNA ladder (GeneRuler, Fermentas); Lane 2, Blank, no RNA in cDNA synthesis. D) Partial sequence chromatogram of the 352 bp cDNA fragment showing that exon 16 of *KAT6A* is fused to exon 2 of *CREBBP*.

46,XY,add(1)(p13),t(8;21)(p11;q22),der(16)t(1;16)(p13;p13)[9]/46,XY[1] (Figure 2A). Four months after diagnosis the bone marrow karyotype was: 47,Y,t(X;17)(p10;q10),der(1)(1qter->1q12::1p22->1q12::5q15->5qter),add(1)(p13),inv(2)(p21q13),der(5)t(1;5)(p22;q15), t(12;18)(q15;q21),+8,t(8;21)(p11;q22),del(13)(q21q31),der(16)t(1;16)(p13;p13)[5]/46,XY[11]. In addition to the primary t(1;16) and t(8;21) translocations, clearly many secondary aberrations had also accrued indicating clonal evolution.

Co-hybridization FISH analysis with the probes RP11-619A23/RP11-95J11 (red, for *CREBBP*) and RP11-642I24/RP11-589C21 (green, for *KAT6A*) revealed a fusion signal of the *KAT6A* and *CREBBP* BACs on the derivative chromosome 8, indicating the presence of a *KAT6A/CREBBP* chimera (Figure 2B). No corresponding fusion signal was found on the der(16)t(1;16) suggesting that the translocation was accompanied by a deletion of the reciprocal *CREBBP/KAT6A* (Figure 2B).

### RT-PCR

PCR with the MOZ3558F and CBP431R primer combination amplified a 352 bp fragment from the patients cDNA (Figure 2C). To verify the presence of a *KAT6A-CREBBP* chimeric transcript, the fragment was analyzed by direct sequencing which showed the presence in the patients bone marrow of a type 1 *KAT6A-CREBBP* chimeric transcript, i.e., the nt 3764 of mRNA of *KAT6A* (accession number NM_006766.3) was fused in-frame with the nt 290 of mRNA of *CREBBP* (accession number NM_004380.2) (Figure 2D).

### RNA-Seq

Using FusionMap on the raw sequencing data obtained from the Genomics Core Facility, 874 fusion transcripts were found (Table S1). The *KAT6A-CREBBP* fusion transcript was not among them (Table S1). Instead, three other *KAT6A* (referred to as *MYST3* in the FusionMap output) fusions were found: *DTX3L-MYST3* ranking 91, *MYST3-SLK* ranking 193, and *MYST-DNAJC14* ranking 606. Based on the map information on the genes, these fusions would have corresponded to the translocations t(3;8)(q21;p11), t(8;10)(p11;q24), and t(8;12)(p11;q13), respectively, none of which was seen by karyotyping. One *CREBP* fusion transcript was found, *CREBBP-TTC28* which was ranked 401 in the list of fusion transcripts (Table S1). The transcript would have corresponded to a t(16;22)(p13;q12) which was not found by G-banding. The FusionFinder program detected 35 fusion transcripts none of which was *KAT6A-CREBBP* (Table S2).

Sequences which contained the first 20 nt of exon 2 of *CREBBP* (ATTTTGGATCATTGTTTGAC; nt 290–319 in sequence with accession number NM_004380.2) were retrieved from the raw sequencing data using the “grep” command. A total of 26 sequences were found (Table 1): 11 of them were *KAT6A-CREBBP* fusions, 15 sequences were exons 1–2 of *CREBBP*, and one was a genomic sequence from the chromosome band 16p13 containing the exon 2 of *CREBBP*. Similar to the results obtained by RT-PCR/Sanger sequencing, all 11 retrieved sequences showed fusion of 3764 nt of mRNA from *KAT6A* (accession number NM_006766.3) with nt 290 of mRNA from *CREBBP* (accession number NM_004380.2) (Table 1).

**Table 1:** The 26 retrieved sequences from the raw sequencing data which contained the first 20ATTTTGGATCATTGTTTGAC (in bold) of *CREBBP*.

RETRIEVED SEQUENCES	*KAT6A* (NM_006766.3)	*CREBBP* (NM_004380.2)
CCCAAAAGAGCCAAACTCAGCTCGCCCGGTTTCTCGGCGAATGACAGCACAG**ATTTTGGATCATTGTTTGAC**TTGGAAAATGATCTTCCTGATGAGCTGAT		238–338 (exon 1–2)
CGGCGAATGACAGCACAG**ATTTTGGATCATTGTTTGAC**TTGGAAAATGATCTTCCTGATGAGCTGATACCCAATGGAGGAGAATTAGGCCTTTTAAACAGT		272–372 (exon 1–2)
*AAGAAGAAGATGAAGAGTCAGATGATGCTGATG* **ATTTTGGATCATTGTTTGAC**TTGGAAAATGATCTTCCTGATGAGCTGATACCCAATGGAGGAGAATTA	3732–3764 (exon 16)	290–357 (exon 2)
ACTTGCTGGACGGACCGCCCAACCCCAAAAGAGCCAAACTCAGCTCGCCCGGTTTCTCGGCGAATGACAGCACAG**ATTTTGGATCATTGTTTGAC**TTGGAA		215–315 (exon 1–2)
*ATGCTGATG* **ATTTTGGATCATTGTTTGAC**TTGGAAAATGATCTTCCTGATGAGCTGATACCCAATGGAGGAGAATTAAGATCGGAAGAGCGTCGTGTAGGG	3756–3764 (exon 16)	290–357 (exon 2)
*GTCAGATGATGCTGATG* **ATTTTGGATCATTGTTTGAC**TTGGAAAATGATCTTCCTGATGAGCTGATACCCAATGGAGGAGAATTAGGCCTTTTAAACAGTG	3748–3764 (exon 16)	290–373 (exon 2)
*AAGAGTCAGATGATGCTGATG* **ATTTTGGATCATTGTTTGAC**TTGGAAAATGATCTTCCTGATGAGCTGATACCCAATGGAGGAGAATTAGGCCTTTTAAAC	3744–3764 (exon 16)	290–369 (exon 2)
*AGAGTCAGATGATGCTGATG* **ATTTTGGATCATTGTTTGAC**TTGGAAAATGATCTTCCTGATGAGCTGATACCCAATGGAGGAGAATTAGGCCTTTTAAACA	3745–3764 (exon 16)	290–370 (exon 2)
AGAACTTGCTGGACGGACCGCCCAACCCCAAAAGAGCCAAACTCAGCTCGCCCGGTTTCTCGGCGAATGACAGCACAG**ATTTTGGATCATTGTTTGAC**TTG		212–312 (exon 1–2)
GACAGCACAG**ATTTTGGATCATTGTTTGAC**TTGGAAAATGATCTTCCTGATGAGCTGATACCCAATGGAGGAGAATTAGGCCTTTTAAACAGTGGGAACCT		280–380 (exon 1–2)
AACTCAGCTCGCCCGGTTTCTCGGCGAATGACAGCACAG**ATTTTGGATCATTGTTTGAC**TTGGAAAATGATCTTCCTGATGAGCTGATACCCAATGTAGGA		251–351 (exon 1–2)
CTGGACGGACCGCCCAACCCCAAAAGAGCCAAACTCAGCTCGCCCGGTTTCTCGGCGAATGACAGCACAG**ATTTTGGATCATTGTTTGAC**TTGGAAAATGA		220–320 (exon 1–2)
AGCCAAACTCAGCTCGCCCGGTTTCTCGGCGAATGACAGCACAG**ATTTTGGATCATTGTTTGAC**TTGGAAAATGATCTTCCTGATGAGCTGATACCAGATC		246–341 (exon 1–2)
*GAAGATGAAGAGTCAGATGATGCTGATG* **ATTTTGGATCATTGTTTGAC**TTGGAAAATGATCTTCCTGATGAGCTGATACCCAATGGAGGGCAGATCGGAAG	3737–3764 (exon 16)	290–350 (exon 2)
GCCAAACTCAGCTCGCCCGGTTTCTCGGCGAATGACAGCACAG**ATTTTGGATCATTGTTTGAC**TTGGAAAATGATCTTCCTGATGAGCTGATACCCAATGG		247–347 (exon 1–2)
*CTCAGGTGTCAGTCCTCTTCTAAGAGGAAGTCTAAAGATGAAGAAGAAGATGAAGAGTCAGATGATGCTGATG* **ATTTTGGATCATTGTTTGAC**TTGGAAAA	3692–3764 (exon 16)	290–317 (exon 2)
CTTGCTGGACGGACCGCCCAACCCCAAAAGAGCCAAACTCAGCTCGCCCGGTTTCTCGGCGAATGACAGCACAG**ATTTTGGATCATTGTTTGAC**TTGGAAA		216–316 (exon 1–2)
AAAGAGCCAAACTCAGCTCGCCCGGTTTCTCGGCGAATGACAGCACAG**ATTTTGGATCATTGTTTGAC**TTGGAAAATGATCTTCCTGATGAGCTGATACCC		242–342 (exon 1–2)
*CAGTCCTCTTCTAAGAGGAAGTCTAAAGATGAAGAAGAAGATGAAGAGTCAGATGATGCTGATG* **ATTTTGGATCATTGTTTGAC**TTGGAAAATGATCTTCC	3701–3764 (exon 16)	290–326 (exon 2)
GCCAAACTCAGCTCGCCCGGTTTCTCGGCGAATGACAGCACAG**ATTTTGGATCATTGTTTGAC**TTGGAAAATGATCTTCCTGATGAGCTGATACCCAATGG		247–347 (exon 1–2)
AAAAGAGCCAAACTCAGCTCGCCCGGTTTCTCGGCGAATGACAGCACAG**ATTTTGGATCATTGTTTGAC**TTGGAAAATGATCTTCCTGATGAGCTGATACC		241–341 (exon 1–2)
GCCAAACTCAGCTCGCCCGGTTTCTCGGCGAATGACAGCACAG**ATTTTGGATCATTGTTTGAC**TTGGAAAATGATCTTCCTGATGAGCTGATACCCAGATC		247–343 (exon 1–2)
*GATGTACTCAGGTGTCAGTCCTCTTCTAAGAGGAAGTCTAAAGATGAAGAAGAAGATGAAGAGTCAGATGATGCTGATG* **ATTTTGGATCATTGTTTGAC**TT	3686–3764 (exon 16)	290–311 (exon 2)
*ATGATGCTGATG* **ATTTTGGATCATTGTTTGAC**TTGGAAAATGATCTTCCTGATGAGCTGATACCCAATGGAGGAGAATTAGGAGATCGGAAGAGCGTCGTG	3753–3764 (exon 16)	290–359 (exon 2)
GTAAAGGTTGCTTAGTTTCTCATTTCCATTTCTGTTTAATTTCTAG**ATTTTGGATCATTGTTTGAC**TTGGAAAATGATCTTCCTGATGAGCTGATACCCAA		290–344 (exon 2)
*GATGATGCTGATG* **ATTTTGGATCATTGTTTGAC**TTGGAAAATGATCTTCCTGATGAGCTGATACCCAATGGAGGAGAATTAGGCCTTTTAAACAGTGGGAA	3752–3764 (exon 16)	290–377 (exon 2)

*KAT6A* sequences are in italics.

## Discussion

The present case of AML had hematologic features highly suggestive of AML with t(8;16)(p11;p13) and a *KAT6A-CREBBP* fusion gene. The leukemic karyotype at diagnosis had two chromosome translocations, a der(16)t(1;16)(p13;p13) and a t(8;21)(p11;q22), indicating the possible generation of *KAT6A-CREBBP* via a cryptic aberration since both chromosome bands 8p11, which contains the *KAT6A* gene, and 16p13, where *CREBBP* maps, were seen rearranged. However, all the chromosome breakpoints in this particular karyotype contain also other genes known to be involved in leukemogenesis. *RBM15* in 1p13 is fused to *MKL1* in AML with t(1;22)(p13;q13) [32], [33]. Likewise, chromosome band 16p13 contains, apart from *CREBBP*, also the *CBFB* gene which is fused to *MYH11* in the subset of AML with inv(16)(p13q22) [34] as well as *GLIS2* which is a partner in the fusion *CBFA2T3-GLIS2* generated by inv(16)(p13q24) [35]. On 8p11, apart from *KAT6A*, *FGFR1* is rearranged in fusions with several partner genes in the “8p11 myeloproliferative syndrome” [36], [37]. For example, the fusion genes *ZNF198-FGFR1*, *CEP110-FGFR1*, *FOP-FGFR1*, and *BCR-FGFR1* result from the t(8;13)(p11;q12), t(8;9)(p11;q33), t(6;8)(q27;p11), and t(8;22)(p11q22) chromosome translocations, respectively [36], [37]. On 21q22, *RUNX1* and *ERG* are fused to *RUNX1T1* and *FUS* generating the *RUNX1-RUNX1T1* and *FUS-ERG* fusion genes in AMLs carrying t(8;21)(p11;q22) and t(16;21)(p11;q22), respectively. In addition, both t(1;16)(p13;p13) and t(8;21)(p11;q22) could conceivably have generated novel leukemogenic fusion genes. Screening with FISH for all possibly rearranged genes associated with the present abnormal karyotype would have been a very laborious and time-consuming procedure. We therefore decided to perform two parallel investigations on the patients bone marrow. Based on the clinical and hematologic hunch that a *KAT6A-CREBBP* fusion was likely, we took the “conventional” approach - karyotyping and FISH followed by RT-PCR - to search for this known leukemogenic gene. In addition, we also performed RNA-Seq to search for this and other possible fusions concentrating exclusively on those fusion transcripts that had something to do with the chromosomal breakpoints. The FISH analysis showed a fusion signal for *KAT6A*- and *CREBBP*- specific probes on the derivative chromosome 8, indicating the presence here of a *KAT6A/CREBBP* chimera (Figure 2B). RT-PCR analysis followed by Sanger sequencing confirmed the presence of type 1 fusion *KAT6A/CREBBP* transcript [15] (Figures 2C and 2D). The transcript retains the part of the *KAT6A* gene encoding the C4HC3 and C2HC zinc fingers, two nuclear localization signals, the HAT domain, the MYST domain, and a portion of the acidic domain, whereas the retained part of *CREBBP* encodes a domain which binds to nuclear receptor RARA, the CREB-binding domain, the three cystein/histidine rich regions, the bromodomain, and the glutamine-rich domains [11].

Surprisingly, of the 874 fusion transcripts identified by the FusionMap program [30], none was the biologically important *KAT6A-CREBBP*, nor was *KAT6A-CREBBP* featured in the list of 35 fusion genes obtained using FusionFinder [31]. Moreover, none of the two programs offered other putative fusion genes generated by the translocations der(16)t(1;16)(p13;p13) and t(8;21)(p11;q22). To find out whether the raw sequencing data contained sequences which encompassed the junction between *KAT6A* and *CREBBP*, we retrieved sequences containing the first 20 nt of exon 2 of *CREBBP* from the raw sequencing data. The rationale behind this was that exon 2 is fused to *KAT6A* (Figures 2C and 2D). Thus, the retrieved sequences should contain both *KAT6A-CREBBP* fusion transcript and wild type *KAT6A-CREBBP* transcript. Indeed, among the altogether 26 retrieved sequences, 11 were *KAT6A-CREBBP* fusions whereas 15 sequences were exons 1-2 of *CREBBP* (Table 1).

Both FusionMap and FusionFinder are among the most commonly used programs to detect fusion genes from RNA-Seq [19] and their sensitivity and specificity have been evaluated [19], [30], [31], [38]. We, too, have in previous studies analyzed RNA-Seq data using the FusionMap program finding that it identified fusion genes that corresponded well with the available cytogenetic information and that were biologically significant [23], [24], [25], [26], [27], [28]. The present study, on the other hand, shows that although the same approach detected hundreds (FusionMap) or tens (FusionFinder) of fusion genes, the programs failed to detect the biologically important *KAT6A-CREBBP* fusion gene although it was manually retrievable from the raw sequencing data.

The case illustrates that RNA-Seq with use of the FusionMap or FusionFinder programs may not be reliable as a stand-alone technique in the investigation of, at least, leukemias. Not only are there far too many false positives offered as fusion genes by this approach, but it may also fail to detect the truly important fusion gene; in the present case neither specificity nor sensitivity was satisfactory. Additional information about clinical, morphological, and cytogenetic features should be taken into account when searching for the crucial fusion genes in hematologic malignancies.

## Supporting Information

Table S1 Identified fusion genes using FusionMap on the raw sequencing data.
(XLSX)

Table S2 Identified fusion genes using FusionFinder.
(XLSX)
